# Gender, anthropometric factors and risk of colorectal cancer with particular reference to tumour location and TNM stage: a cohort study

**DOI:** 10.1186/2042-6410-3-23

**Published:** 2012-10-16

**Authors:** Jenny Brändstedt, Sakarias Wangefjord, Björn Nodin, Alexander Gaber, Jonas Manjer, Karin Jirström

**Affiliations:** 1 Department of Clinical Sciences, Division of Pathology, Lund University, Skåne University Hospital, Lund, SE-221 85, Sweden; 2 Department of Clinical Sciences, Division of Surgery, Lund University, Skåne University Hospital, Malmö, SE-205 02, Sweden; 3 The Malmö Diet and Cancer Study, Lund University, Malmö, SE-205 02, Sweden

## Abstract

**Background:**

It remains unclear whether the increased risk of colorectal cancer (CRC) associated with obesity differs by gender, distribution of fat, tumour location and clinical (TNM) stage. The primary aim of this study was to examine these associations in 584 incident colorectal cancer cases from a Swedish prospective population-based cohort including 28098 men and women.

**Methods:**

Seven anthropometric factors; height, weight, bodyfat percentage, hip circumference, waist circumference, BMI and waist-hip ratio (WHR) were categorized into quartiles of baseline anthropometric measurements. Relative risks of CRC, total risk as well as risk of different TNM stages, and risk of tumours located to the colon or rectum, were calculated for all cases, women and men, respectively, using multivariate Cox regression models.

**Results:**

Obesity, as defined by all anthropometric variables, was significantly associated with an overall increased risk of CRC in both women and men. While none of the anthropometric measures was significantly associated with risk of tumour (T)-stage 1 and 2 tumours, all anthropometric variables were significantly associated with an increased risk of T-stage 3 and 4, in particular in men. In men, increasing quartiles of weight, hip, waist, BMI and WHR were significantly associated with an increased risk of lymph node positive (N1 and N2) disease, and risk of both non-metastatic (M0) and metastatic (M1) disease. In women, there were no or weak associations between obesity and risk of node-positive disease, but statistically significant associations between increased weight, bodyfat percentage, hip, BMI and M0 disease. Interestingly, there was an increased risk of colon but not rectal cancer in men, and rectal but not colon cancer in women, by increased measures of weight, hip-, waist circumference and bodyfat percentage.

**Conclusions:**

This study is the first to show a relationship between obesity, measured as several different anthropometric factors, and an increased risk of colorectal cancer of more advanced clinical stage, in particular in men. These findings suggest that risk of CRC differs according to the method of characterising obesity, and also according to gender, location, and tumour stage.

## Background

Colorectal cancer (CRC) is the third most common malignancy in developed countries with approximately 1 million new cases every year [1]. Life style factors may contribute to the aetiology of several cancer forms [2], including CRC and, since they are modifiable, a better understanding of the associations between lifestyle-related factors and CRC risk may be of importance in order to develop novel strategies for prevention of the disease.

Numerous epidemiological studies and meta-analyses have examined the relationship between body weight and body mass index (BMI) and CRC, mainly risk of colon cancer, and most studies have shown a positive relationship between a high BMI and risk of colon cancer in men, whereas weak or no associations were reported in women [3-16]. The reasons for the apparently discrepant associations between BMI and CRC risk in men and women remain unclear. Due to inconsistencies in the definition of obesity, the results of epidemiologic studies may be somewhat difficult to generalize, although the majority of studies have used BMI as the principal measure of adiposity. The discrepancy between men and women in the association of body weight with CRC may be related to differences in fat distribution between the sexes. A high BMI in men is more closely related to central/abdominal obesity, whereas in women, a high BMI more often correlates with lower body obesity, measured as hip circumference [17]. Some prospective studies have investigated the association of body fat distribution measured as waist-, hip circumference and waist-hip ratio (WHR) and CRC risk [6,8,12,13,18-23]. All of these studies found waist circumference to be associated with an increased risk. However, in most of these studies, waist- and hip measurements were self-reported rather than measured [8,20,23]. One large study [6] has shown that both waist circumference and WHR are strongly related to CRC risk in both men and women, supporting the hypothesis that abdominal obesity is a risk factor for CRC in both sexes and suggesting that fat distribution is more important than body weight or BMI in women.

A small number of studies have examined associations of other anthropometric factors than BMI and CRC risk according to tumour location; i.e. colon or rectum [4,6,14,22]. Most of these studies found no association with body weight or BMI according to location, while one study has shown an increased risk of rectal cancer in overweight/obese men [14].

To our knowledge, only two previous studies [13,18] have addressed the question of whether obesity is associated with an increased risk of developing CRC of different clinical (TNM) stages, based on the extent of the tumour (T), the extent of spread to the lymph nodes (N), and the presence of distant metastasis (M). The aim of this study was therefore to examine these associations in participants in the Malmö Diet and Cancer Study (MDCS), a large prospective population based Swedish cohort study. A further aim was to examine whether these associations differ between men and women.

## Methods

### The Malmö Diet and Cancer Study

The Malmö Diet and Cancer Study (MDCS) is a population-based prospective cohort study, forming part of the European Prospective Investigation into Cancer and Nutrition (EPIC)[24] that enrolled 28098 individuals during baseline examinations between 1991 and 1996 [25].

The main purpose of the MDCS was to further investigate the associations between a Western diet rich in fat and low in fruit and vegetables and certain forms of cancer. Participants in the MDCS and EPIC cohort include 11063 men (39.4%) and 17035 women (60.6%) between 44 and 74 years of age. Data on lifestyle, health and socio-demographic characteristics were collected via standardized self-administered questionnaires. Ethical permission for the MDCS (LU 90-51) and the present study (LU 530-2008) was obtained from the Ethics Committee at Lund University.

### Anthropometric measurements

At baseline examination, weight (multiples of 0.1 kg) and height (to the nearest 0.005 m) was measured by a trained nurse, and body mass index (BMI) was calculated as kg/m^2^. Waist circumference was measured at the midpoint between the lower ribs and the iliac crest, and for hip circumference the level of greatest lateral extension was used. These measurements were estimated to the nearest 0.01 m. The waist and hip circumferences of each participant were used to calculate waist-hip ratio (WHR; cm/cm) as an additional measure of fat distribution. Body composition was estimated using a single frequency bio-impedance methodology (BIA 103, RLJ-systems, Detroit, MI, USA) with tetra polar electrode placement and subjects in a supine position. Lean body mass and fat mass were determined and served to calculate body fat percentage. The BIA method has previously been validated in Swedish middle-aged and elderly adults [26].

### Follow-up for cancer and cause-of-death

Incident cases of invasive colorectal cancer in the MDCS were identified through the Swedish Cancer Registry and vital status was determined by record linkage with the Swedish Cause-of -Death Registry. End of follow-up was 31 December 2009. Information on vital status and cause of death was obtained from the Swedish Cause of Death Registry until 31 December 2009. Time on study was defined as time from baseline to diagnosis, death or end of follow-up 31 December 2009. Median time from baseline until diagnosis was 8.6 (SD = 4.3) years and the median follow-up time in the entire cohort was 13.7 (SD = 3.2) years.

### Study population

Among the 28098 men and women in the cohort, there was a total number of 584 cases of incident colorectal cancer. Eight tumours were re-classified as tumour in situ (TIS), and these were not included as cases. A total number of 181 cases were diagnosed with CRC before baseline examination, i.e prevalent colorectal cancers, and therefore excluded from the study. Cases with other prevalent cancers were not excluded from the study.

### Tumour characteristics

Patient and tumour characteristics in the cohort have been described in detail previously [27-30]. The distribution of clinicopathological characteristics did not differ between all CRC cases in the MDCS (n = 626) and cases included in the EPIC cohort (n = 584) (data not shown). Of all incident CRC cases, 337 (59%) had tumours located in the colon and 216 (38%) had tumours located in the rectum. Cases with multiple synchronous location (n = 14), and cases with unknown location (n = 17) were excluded from the analysis related to tumour location. All tumours were histopathologically re-evaluated by a senior pathologist (KJ). According to the TNM classification, 51 (9.7%) cases presented in T-stage 1, 62 (11.8%) in T-stage 2, 323 (61.3%) in T-stage 3 and 82 (15.6%) in T-stage 4. Two hundred and ninety two (58.4%) cases presented with lymph node negative (N0) disease, 124 (24.8%) had N1 (1-3 positive lymph nodes) and 84 (16.8%) N2 (4 or more positive lymph nodes) disease. Four hundred and fifty (79.5%) patients did not have distant metastases (M0), and 116 (20.4%) had M1 disease. The distribution of clinicopathological characteristics and prognostic factors as well as adjuvant and palliative treatment did not differ between men and women in the cohort [27].

### Statistical methods

Distribution of established and potential risk factors for CRC was compared between CRC cases and the rest of the study cohort. Anthropometric measurements were divided into quartiles. Separate quartiles were calculated for men and women for this analysis (Additional file 1). A Cox proportional hazards analysis was used in order to compare risk of colorectal cancer between different categories of anthropometric factors in both sexes, and for women and men separately. This yielded relative risks (RR) with a 95% confidence interval. Time on study was used as the underlying time scale, defined as time from baseline to diagnosis, death or end of follow-up 31 December 2009. The proportional hazards assumption was confirmed by a log, - log plot [31]. In the multivariate Cox analysis potential confounders were included, i.e age (years), educational level (not completed school/elementary school (6-8 years)/ “grundskola” (9-10 years)/ “studentexamen” (10-12 years)/ one year after “studentexamen”/ university degree), smoking habits (yes regularly, yes occasionally, former smoker, never smoker), and alcohol consumption (g/day) (Table 1). All statistical analyses were conducted using SPSS version 16 (SPSS Inc., Chicago, IL, USA). A two-tailed p-value less than 0.05 was regarded as statistically significant.

**Table 1:** Distribution of risk factors in cases and rest of cohort

**Factor (number of subjects with information)**	** Category**	**CRC cases (n = 584)**	**Cases, men (n = 280)**	**Cases, women (n = 304)**	**Rest of cohort (n = 27514)**	**Men (n = 10783)**	**Women (n = 16731)**
		Percent* (mean and SD in italics)					
Age at baseline	years	61.8*(6.8)*	61.7*(6.7)*	62.1*(6.8)*	58.0*(7.6)*	59.2*(7.1)*	57.2*(7.9)*
Education (28027)	Not completed school	0.9	0.7	0.9	0.8	0.8	0.8
	6-8 years	49.1	48.5	46.8	41.0	45.0	36.1
	9-10 years	22.4	19.6	22.8	26.2	19.6	28.6
	10-12 years	8.7	10.4	6.8	8.9	11.9	6.6
	1 year university	7.7	6.8	8.3	8.7	9.3	7.9
	University degree	10.8	13.6	7.7	14.3	13.2	14.2
Smoking status (28086)	Regularly	21.2	18.9	23.4	23.8	23.9	22.5
	Occasionally	3.4	4.3	2.6	4.5	4.8	4.1
	Former smoker	39.7	51.8	28.6	33.7	43.0	26.2
	Never smoked	35.6	25.0	45.4	37.9	28.3	41.7
Alcohol consumption (28098)	g/day	*10.8(14.2)*	*15.7/17.4)*	*6.2(8.1)*	*10.7(12.7)*	*15.5(16.0)*	*7.7(8.7)*
Heigh (28057)t	cm	*169.5(8.9)*	*176.3(6.4)*	*163.3(5.5)*	*168.6(8.8)*	*176.4(6.6)*	*163.6(6.1)*
Weight (28056)	kg	*76.6(14.2)*	*83.8(12.6)*	*70.0(12.2)*	*73.4(13.6*	*81.7(12.1)*	*68.0(11.7)*
Bodyfat percentage (27925)	%	*26.9(7.1)*	*21.5(5.0)*	*31.9(4.8)*	*26.8(7.0)*	*20.7(5.0)*	*30.8(5.0)*
Hip (28044)	cm	*100.7(9.0)*	*101.2(7.4)*	*100.2(10.2)*	*98.4(8.8)*	*99.3(7.1)*	*97.8(9.9)*
Waist (28045)	cm	*87.9(13.7)*	*96.3(10.7)*	*80.1(11.3)*	*84.1(14.5)*	*93.7(12.5)*	*77.9(12.1*
Bodymass index (28056)	kg/m²	*26.6(4.0)*	*26.9(3.6)*	*26.3(4.4)*	*25.7(4.0)*	*26.2(3.5)*	*25.4(4.2)*
Waist-hip ratio (28043)	cm/cm	*0.87(0.09)*	*0.95(0.06*	*0.80(0.05)*	*0.85(0.14)*	*0.94(0.10*	*0.80(0.14)*

## Results

### Baseline characteristics

The distribution of covariates and mean values for the anthropometric measures for men and women who developed CRC during follow-up (cases), and for those who did not (rest of cohort) is presented in Table 1. Cases were slightly older than the rest of cohort, and men were generally older than women in the rest of cohort, but among cases men were slightly younger than women. Mean value for all anthropometric factors were higher in cases as compared to the rest of cohort. Among cases, 47.9% were men, and 52.1% women.

### Overall risk of colorectal cancer in relation to anthropometric factors

As shown in Table 2, there was a statistically significant association between all anthropometric factors except bodyfat percentage and an increased risk of CRC in both sexes. In men, strong, statistically significant associations were seen for all factors except height. In women, BMI, hip and weight, but not the other anthropometric measures, were associated with an increased risk of CRC.

**Table 2:** Anthropometric factors and relative risk of colorectal cancer in all cases, women and men

**Anthropometric factor (quartiles)**	**(n)**	** RR all n = 584**	**(n)**	** RR men n = 280**	**(n)**	** RR women n = 304**
Height	127	1.00	60	1.00	58	1.00
	130	0.99(0.77-1.27)	70	1.28(0.91-1.82)	99	1.24(0.89-1.71)
	154	1.17(0.92-1.49)	75	1.08(0.77-1.52)	77	1.33(0.95-1.88)
	173	1.28(1.00-1.63)	75	1.28(0.90-1.82)	68	1.29(0.89-1.85)
*p-trend*		*0.021*		*0.327*		*0.160*
Weight	108	1.00	52	1.00	45	1.00
	130	1.23(0.95-1.59)	67	1.22(0.85-1.76)	83	1.58(1.10-2.27)
	150	1.21(0.94-1.56)	67	1.25(0.87-1.80)	83	1.83(1.27-2.65)
	196	1.61(1.26-2.05)	94	1.78(1.26-2.52)	92	1.68(1.17-2.41)
*p-trend*		*<0.001*		*0.001*		*0.007*
Bodyfatpercentage	131	1.00	41	1.00	51	1.00
	145	1.07(0.84-1.36)	58	1.10(0.74-1.64)	58	1.09(0.74-1.59)
	122	0.90(0.70-1.16)	91	1.25(0.87-1.82)	81	1.33(0.93-1.90)
	186	1.06(0.84-1.34)	88	1.54(1.06-2.23)	113	1.30(0.92-1.82)
*p-trend*		*0.858*		*0.012*		*0.086*
Hip	96	1.00	47	1.00	45	1.00
	118	1.03(0.79-1.36)	54	1.08(0.73-1.60)	70	1.11(0.76-1.62)
	170	1.43(1.11-1.84)	86	1.65(1.15-2.36)	82	1.27(0.88-1.84)
	200	1.41(1.10-1.81)	93	1.48(1.03-2.11)	106	1.39(0.97-1.99)
*P-trend*		*0.001*		*0.007*		*0.042*
Waist	94	1.00	52	1.00	44	1.00
	140	1.31(1.01-1.71)	44	0.75(0.50-1.12)	71	1.15(0.79-1.69)
	125	1.03(0.78-1.35)	84	1.32(0.93-1.88)	99	1.46(1.02-2.08)
	225	1.76(1.37-2.27)	100	1.65(1.17-2.31)	89	1.29(0.89-1.86)
*P- trend*		*<0.001*		*<0.001*		*0.114*
BMI	99	1.00	64	1.00	48	1.00
	124	1.25(0.96-1.63)	49	0.73(0.50-1.06)	82	1.53(1.07-2.19)
	162	1.22(0.94-1.57)	67	0.94(0.67-1.33)	81	1.43(1.00-2.05)
	199	1.69(1.32-2.17)	100	1.61(1.17-2.22)	92	1.61(1.12-2.30)
*P-trend*		*<0.001*		*0.001*		*0.027*
WHR	124	1.00	66	1.00	77	1.00
	127	1.02(0.79-1.30)	47	0.99(0.68-1.44)	65	1.02(0.73-1.42)
	145	1.06(0.83-1.36)	81	1.36(0.98-1.88)	81	1.04(0.76-1.43)
	187	1.49(1.17-1.89)	86	1.64(1.19-2.28)	80	1.14(0.83-1.57)
*P-trend*		*0.001*		*0.001*		*0.413*

Adjusted for age at baseline, level of education, smoking habits, alcohol consumption.

### Relative risk of colorectal cancer in relation to T, N and M-stage

As shown in Table 3, there were no associations between any of the anthropometric measures and T-stage 1 and 2. However, weight, hip, waist, BMI and WHR were strongly associated with an increased risk of tumours with T-stage 3 and 4 in both sexes (Table 3). The associations between obesity and more advanced tumour stage (T-stage 3 and 4) were more evident in men (Tables 4 and 5), with a significant relationship between all anthropometric factors, except height, and more advanced T-stage in men, while this relationship was only significant for weight, bodyfat percentage and BMI in women (Tables 4 and 5). Regarding N-stage, there was an overall increased risk of N1 and N2-tumours for all anthropometric factors except bodyfat percentage and height (Table 3). Subgroup analysis according to gender revealed that these associations were only statistically significant in men (Table 4) and not, except for bodyfat percentage, in women (Table 5). The overall associations between obesity and M-stage were stronger for M0 compared to M1 disease (Table 3). Similar findings were seen among women, where no anthropometric factors were associated with M1 disease (Table 4), while in men, weight, waist and WHR were statistically significant associated with both M0 and M1 disease (Table 4).

**Table 3:** Anthropometric factors and relative risk of colorectal cancer of different T, N- and M stages in all cases

**Anthropometric factor (quartiles)**	**(n)**	**RR of T-stage 1&2 n=113**	**(n)**	**RR of T-stage 3&4 n=405**	**(n)**	**RR of N-stage 0 n=292**	**(n)**	**RR of N-stage 1&2 n=208**	**(n)**	**RR of M-stage 0 n=450**	**(n)**	**RR of M-stage 1 n=116**
Height	25	1.00	91	1.00	61	1.00	46	1.00	92	1.00	35	1.00
	26	0.97(0.56-1.68)	92	1.00(0.74-1.33)	68	1.10(0.78-1.57)	49	0.99(0.66-1.49)	109	1.12(0.83-1.47)	18	0.51(0.29-0.91)
	33	1.00(0.58-1.74)	102	1.12(0.83-1.49)	83	1.33(0.95-1.87)	38	0.80(0.51-1.23)	118	1.12(0.84-1.49)	26	0.76(0.45-1.28)
	29	1.06(0.61-1.85)	120	1.29(0.96-1.72)	80	1.24(0.87-1.77)	75	1.52(1.03-2.24)	132	1.32(0.98-1.78)	37	1.02(0.62-1.68)
*p-trend*		*0.807*		*0.060*		*0.153*		*0.055*		*0.037*		*0.678*
Weight	17	1.00	77	1.00	60	1.00	37	1.00	80	1.00	26	1.00
	38	2.21(1.24-3.93)	78	1.03(0.75-1.42)	66	1.11(0.78-1.58)	40	1.12(0.72-1.76)	111	1.09(0.84-1.41)	16	0.64(0.35-1.20)
	26	1.26(0.68-2.34)	109	1.24(0.92-1.66)	71	1.00(0.71-1.42)	55	1.34(0.88-2.03)	109	1.23(0.94-1.61)	36	1.22(0.73-2.04)
	32	1.52(0.83-2.78)	141	1.64(1.23-2.18)	95	1.35(0.97-1.89)	76	1.87(1.25-2.80)	151	1.61(1.25-2.09)	38	1.31(0.78-2.20)
*p-trend*		*0.673*		*<0.001*		*0.116*		*0.001*		*0.002*		*0.100*
Body fat percentage	25	1.00	86	1.00	71	1.00	37	1.00	96	1.00	31	1.00
	27	1.10(0.61-1.82)	104	1.17(088-1.56)	67	0.92(0.66-1.29)	61	1.56(1.04-2.35)	115	1.13(0.72-1.79)	28	0.88(0.53-1.46)
	25	1.04(0.59-1.84)	82	0.92(0.67-1.25)	61	0.86(0.61-1.22)	42	1.07(0.68-1.67)		1.24(0.82-1.88)	24	0.75(0.44-1.30)
	36	1.21(0.71-2.06)	133	1.13(0.85-1.50)	93	0.99(0.72-1.37)	68	1.36(0.90-2.05)	91	1.17(0.80-1.70)		0.76(0.45-1.27)
*p-trend*		*0.498*		*0.706*		0.948		*0.460*	149	*0.483*	33	*0.261*
Hip	19	1.00	65	1.00	56	1.00	30	1.00	74	1.00	22	1.00
	21	0.92(0.49-1.71)	53	1.02(0.74-1.42)	53	0.77(0.54-1.15)	41	1.18(0.74-1.90)	93	1.08(0.80-1.44)	18	0.68(0.37-1.27)
	32	1.30(0.73-2.32)	119	1.48(1.09-2.01)	84	1.18(0.84-1.67)	61	1.74(1.12-2.71)	122	1.47(1.11-1.93)	41	1.52(0.90-2.56)
	41	1.46(0.84-2.55)	142	1.46(1.08-1.98)	99	1.17(0.84-1.63)		1.80(1.17-2.77)	162	1.52(1.18-1.95)	35	1.04(0.60-1.79)
*p-trend*		*0.077*		*0.002*		0.089		*0.002*		*0.001*		*0.323*
Waist	19	1.00	62	1.00	49	1.00	33	1.00	70	1.00	21	1.00
		1.45(0.81-2.60)	91	1.28(0.93-1.78)	71	1.23(0.85-1.78)	45	1.23(0.79-1.94)	109	0.81(0.60-1.09)	23	0.95(0.52-1.71)
	31	0.98(0.53-1.81)	85	1.06(0.76-1.48)	57	0.85(0.58-1.26)	44	1.09(0.69-1.73)	97	1.38(1.07-1.79)	25	0.90(0.50-1.63)
	25	1.36(0.75-2.43)	167	2.00(1.49-2.72)	115	1.60(1.13-2.27)	86	2.10(1.38-3.18)	175	1.70(1.32-2.19)	47	1.63(0.96-2.80)
*p-trend*	38	*0.596*		*<0.001*		*0.022*		*0.001*		*<0.001*		*0.049*
BMI	22	1.00	64	1.00	51	1.00	34	1.00	74	1.00	23	1.00
	28	1.22(0.69-2.15)	78	1.22(0.87-1.70)	69	1.33(0.92-1.92)	39	1.18(0.75-1.88)	99	1.19(0.91-1.56)	21	0.93(0.51-1.68)
	32	1.05(0.60-1.81)	111	1.29(0.94-1.75)	73	1.03(0.72-1.48)	59	1.34(0.87-2.65)	127	1.10(0.83-1.45)	29	0.95(0.55-1.65)
	31	1.16(0.66-2.03)	152	1.97(1.46-2.66)	99	1.56(1.10-2.20)	76	1.98(1.31-2.99)	151	1.63(1.27-2.10)	43	1.59(0.95-2.67)
*p-trend*		*0.773*		*<0.001*		*0.043*		*0.001*		*<0.001*		*0.063*
WHR	28	1.00	81	1.00	65	1.00	43	1.00	101	1.00	20	1.00
	25	0.90(0.52-1.56)	87	1.05(0.78-1.43)	58	0.85(0.60-1.22)	48	1.14(0.75-1.72)	91	1.01(0.73-1.41)	29	1.40(0.79-2.48)
	30	0.91(0.54-1.55)	97	1.10(0.81-1.48)	83	1.10(0.79-1.53)	39	0.88(0.56-1.36)	117	1.23(0.92-1.64)	26	1.20(0.66-2.16)
	30	0.89(0.51-1.56)	139	1.74(1.30-2.33)	86	1.19(0.84-1.67)	77	1.95(1.32-2.89)	141	1.58(1.19-2.09)	41	2.10(1.20-3.69)
*p-trend*		*0.707*		*<0.001*		*0.176*		*0.003*		*0.013*		*0.017*

Adjusted for age, level of education, smoking habits and alcohol consumption.

**Table 4:** Anthropometric factors and relative risk of colorectal cancer of different T , N and M stages in men

**Anthropometric factor (quartiles)**	**(n)**	**RR of T-stage 1&2 n=50**	**(n)**	**RR of T-stage 3&4 n=199**	**(n)**	**RR of N-stage 0 n=140**	**(n)**	**RR of N-stage 1&2 n=102**	**(n)**	**RR of M-stage 0 n=214**	**(n)**	**RR of M-stage 1 n=60**
Height	9	1.00	48	1.00	37	1.00	16	1.00	49	1.00	11	1.00
	11	1.24(0.51-3.01)	47	1.09(0.73-1.63)	34	1.02(0.64-1.62)	21	1.42(0.74-2.72)	51	1.14(0.77-1.69)	17	1.75(0.82-3.74)
	18	1.56(0.69-3.49)	47	0.87(0.58-1.31)	31	0.74(0.46-1.21)	33	1.72(0.94-3.14)	58	1.02(0.69-1.50)	13	1.07(0.48-2.40)
	12	1.13(0.47-2.75)	57	1.29(0.87-1.91)	38	1.10(0.69-1.77)	32	1.93(1.04-3.56)	56	1.16(0.78-1.73)	19	1.94(0.90-4.16)
*p-trend*		*0.677*		*0.406*		*0.978*		*0.029*		*0.600*		*0.226*
Weight	13	1.00	33	1.00	32	1.00	11	1.00	43	1.00	9	1.00
	11	0.77(0.34-1.72)	51	1.48(0.95-2.29)	34	1.00(0.62-1.62)	26	2.23(1.10-4.53)	48	1.05(0.69-1.58)	18	2.02(0.90-4.51)
	12	0.82(0.37-1.82)	47	1.41(0.90-2.20)	35	1.06(0.65-1.72)	24	2.06(1.01-4.22)	56	1.25(0.83-1.86)	11	1-31(0.54-3.17)
	14	0.90(0.42-1.96)	68	2.09(1.37-3.19)	39	1.19(0.74-1.91)	41	3.58(1.82-7.01)	67	1.50(1.02-2.22)	22	2.75(1.25-6.04)
*p-trend*		*0.867*		*0.001*		*0.448*		*<0.001*		*0.023*		*0.032*
Body fat percentage	8	1.00	29	1.00	24	1.00	10	1.00	30	1.00	10	1.00
	9	0.89(0.34-2.32)	40	1.07(0.66-1.72)	30	0.96(0.56-1.65)	18	1.41(0.65-3.05)	44	1.13(0.71-1.81)	11	0.87(0.37-2.04)
	19	1.36(0.59-3.12)	62	1.21(0.78-1.88)	43	0.99(0.60-1.63)	36	2.06(1.02-4.17)	70	1.31(0.85-2.01)	20	1.16(0.54-2.48)
	13	1.14(0.47-2.76)	67	1.66(1.07-2.57)	42	1.22(0.74-2.02)	37	2.69(1.33-5.43)	69	1.64(1.06-2.52)	18	1.33(0.61-2.89)
		*0.547*		*0.011*		*0.390*		*<0.001*		*0.013*		*0.332*
Hip	9	1.00	32	1.00	32	1.00	8	1.00	36	1.00	11	1.00
	13	1.36(0.58-3.18)	32	0.95(0.58-1.55)	26	0.77(0.46-1.29)	18	2.12(0.92-4.88)	44	1.15(0.74-1.79)	7	0.62(0.241.60)
	11	1.11(0.46-2.69)	67	1.89(1.23-2.89)	38	1.06(0.66-1.70)	37	4.20(1.95-9.05)	61	1.52(1.00-2.30)	23	1.99(0.96-4.12)
	17	1.37(0.60-3.13)	68	1.58(1.04-2.44)	44	1.02(0.64-1.62)	39	3.65(1.70-7.88)	73	1.50(1.00-2.25)	19	1.39(0.65-2.95)
*p-trend*		*0.577*		*0.003*		*0.619*		*<0.001*		*0.025*		*0.102*
Waist	12	1.00	34	1.00	32	1.00	11	1.00	41	1.00	10	1.00
	11	0.80(0.35-1.83)	27	0.71(0.43-1.17)	20	0.55(0.31-0.96)	16	1.30(0.61-2.81)	33	0.71(0.45-1.12)	9	0.81(0.33-1.99)
	12	0.80(0.36-1.79)	63	1.53(1.00-2.32)	44	1.10(0.70-1.74)	31	2.34(0.65-2.30)	67	1.32(0.90-1.96)	17	1.47(0.67-3.23)
	15	1.00(0.46-2.16)	75	1.90(1.26-2.87)	44	1.13(0.72-1.80)	44	3.51(1.80-6.83)	73	1.50(1.02-2.22)	24	2.18(0.04-4.60)
*p-trend*		*0.966*		*<0.001*		*0.155*		*<0.001*		*0.002*		*0.009*
BMI	16	1.00	39	1.00	37	1.00	17	1.00	50	1.00	14	1.00
	11	0.65(0.30-1.42)	35	0.86(0.54-1.35)	24	0.61(0.36-1.02)	21	1.19(0.63-2.26)	41	0.77(0.51-1.17)	8	0.57(0.24-1.37)
	10	0.54(0.24-1.20)	48	1.11(0.73-1.71)	33	0.78(0.48-1.25)	23	1.22(0.65-2.30)	48	0.85(0.57-1.26)	18	1.27(0.62-2.57)
		0.78(0.37-1.64)	77	2.04(1.38-3.03)	46	1.22(0.79-1.91)	41	2.51(1.41-4.46)	75	1.51(1.05-2.17)	20	1.62(0.80-3.24)
*p-trend*		*0.420*		*<0.001*		*0.218*		*0.001*		*0.015*		*0.058*
WHR	14	1.00	44	1.00	43	1.00	16	1.00	53	1.00	14	1.00
	10	0.96(0.43-2.17)	32	1.02(0.65-1.61)	23	0.78(0.47-1.31)	17	1.49(0.75-2.96)	38	0.99(0.65-1.50)	7	0.71(0.29-1.76)
	18	1.35(0.67-2.73)	55	1.39(0.93-2.07)	34	0.90(0.57-1.43)	35	2.49(1.38-4.51)	59	1.22(0.84-1.77)	19	1.53(0.77-3.06)
	8	0.65(0.27-1.56)	68	1.98(1.35-2.91)	43	1.29(0.83-1.99)	34	2.76(1.52-5.04)	64	1.50(1.03-2.17)	20	1.88(0.94-3.76)
*p-trend*		*0.631*		*<0.001*		*0.251*		*<0.001*		*0.021*		*0.028*

Adjusted for age, level of education, smoking habits and alcohol consumption.

**Table 5:** Anthropometric factors and relative risk of colorectal cancer of different T , N and M stages in women

**Anthropometric factor (quartiles)**	**(n)**	**RR of Tstage 1&2 n=63**	**(n)**	**RR of Tstage 3&4 n=205**	**(n)**	**RR of N-stage 0 n=150**	**(n)**	**RR of N-stage1 & 2 n=105**	**(n)**	**RR of M-stage 0 n=236**	**(n)**	**RR of M-stage 1 n=56**
Height	11	1.00	43	1.00	32	1.00	21	1.00	45	1.00	14	1.00
	20	1.40(0.67-2.94)	69	1.18(0.80-1.73)	49	1.16(0.74-1.82)	33	1.12(0.64-1.93)	74	1.23(0.85-1.79)	24	1.18(0.61-2.31)
	15	1.43(0.65-3.14)	54	1.29(0.86-1.94)	36	1.20(0.74-1.95)	34	1.53(0.88-2.66)	67	1.53(1.04-2.24)	8	0.56(0.23-1.35)
	17	1.81(0.81-4.00)	39	1.02(0.65-1.60)	35	1.33(0.80-2.19)	17	0.81(0.42-1.57)	50	1.23(0.81-1.87)	10	0.77(0.33-1.78)
*p-trend*		*0.168*		*0.787*		*0.274*		*0.925*		*0.184*		*0.233*
Weight	8	1.00	31	1.00	23	1.00	18	1.00	32	1.00	12	1.00
	16	1.71(0.73-4.00)	54	1.49(0.96-2.32)	39	1.45(0.88-2.46)	30	1.40(0.78-2.52)	62	1.66(1.09-2.55)	19	1.34(0.65-2.76)
	22	2.67(1.18-6.07)	54	1.74(1.18-2.72)	43	1.90(1.14-3.16)	27	1.51(0.83-2.74)	71	2.21(1.45-3.37)	7	0.59(0.23-1.49)
	17	1.85(0.79-4.32)	66	1.70(1.10-2.62)	47	1.69(1.02-2.80)	30	1.35(0.75-2.43)	71	1.85(2121-2.81)	18	1.16(0.55-2.45)
*p-trend*		*0.114*		*0.018*		*0.034*		*0.367*		*0.004*		*0.865*
Body fat percentage	11	1.00	28	1.00	26	1.00	14	1.00	37	1.00	10	1.00
	15	1.25(0.57-2.77)	37	1.26(0.77-2.07)	31	1.13(0.67-1.91)	18	1.29(0.64-2.60)	44	1.13(0.72-1.76)	11	1.07(0.45-2.52)
	15	1.11(0.51-2.45)	57	1.71(1.08-2.69)	40	1.25(0.76-2.05)	31	1.98(1.05-3.74)	66	1.50(1.00-2.25)	13	1.09(0.47-2.51)
	22	1.15(0.55-2.42)	83	1.71(1.11-2.66)	55	1.17(0.73-1.90)	42	1.92(1.03(3.57)	89	1.42(0.96-2.11)	22	1.26(0.58-2.72)
*p-trend*		*0.840*		*0.009*		*0.516*		*0.021*		*0.046*		*0.539*
Hip	10	1.00	28	1.00	23	1.00	18	1.00	32	1.00	12	1.00
	11	0.77(0.33-1.83)	49	1.25(0.79-2.00)	36	1.12(0.66-1.90)	22	0.88(0.47-1.65)	56	1.27(0.82-1.97)	11	0.62(0.27-1.41)
	18	1.18(0.53-2.60)	55	1.38(0.87-2.19)	38	1.14(0.67-1.93)	29	1.20(0.66-2.17)	60	1.32(0.85-2.04)	17	0.95(0.45-2.01)
	24	1.44(0.68-3.07)	73	1.51(0.97-2.36)	35	1.38(0.84-2.28)	36	1.24(0.70-2.22)	88	1.67(1.11-2.53)	16	0.70(0.32-1.52)
*p-trend*		*0.149*		*0.066*		*0.184*		*0.259*		*0.012*		*0.641*
Waist	9	1.00	32	1.00	24	1.00	18	1.00	32	1.00	12	1.00
	13	0.95(0.40-2.26)	43	0.97(0.61-1.55)	34	0.99(0.58-1.68)	22	0.93(0.50-1.74)	54	1.20(0.77-1.87)	11	0.67(0.30-1.53)
	22	1.53(0.70-3.34)	67	1.36(0.89-2.08)	47	1.23(0.75-2.01)	37	1.41(0.80-2.50)	79	1.61(1.06-2.43)	16	0.86(0.40-1.83)
	19	1.33(0.59-2.97)	63	1.23(0.80-1.90)	47	1.17(0.71-1.94)	28	1.08(0.59-2.00)	71	1.43(0.94-2.19)	17	0.86(0.40-1.85)
*p-trend*		*0.293*		*0.161*		*0.375*		*0.491*		*0.054*		*0.933*
BMI	10	1.00	30	1.00	25	1.00	16	1.00	35	1.00	11	1.00
	16	1.36(0.61-3.03)	54	1.62(1.03-2.53)	39	1.38(0.83-2.30)	32	1.86(1.02-3.40)	32	1.60(1.06-2.43)	15	1.23(0.57-2.70)
	19	1.58(0.73-3.42)	53	1.49(0.95-2.34)	39	1.30(0.78-2.15)	25	1.38(0.73-2.60)	65	1.58(1.04-2.40)	11	0.83(0.36-1.94)
	18	1.56(0.71-3.45)	68	1.84(1.18-2.86)	49	1.58(0.96-2.60)	32	1.76(0.95-3.25)	73	1.77(1.17-2.67)	19	1.38(0.64-2.98)
*p-trend*		*0.259*		*0.017*		*0.107*		*0.205*		*0.016*		*0.592*
WHR	17	1.00	50	1.00	40	1.00	25	1.00	66	1.00	14	1.00
	15	1.11(0.55-2.24)	41	0.98(0.65-1.48)	35	1.04(0.66-1.64)	22	1.04(0.59-1.85)	54	1.07(0.74-1.54)	9	0.78(0.34-1.80)
	15	0.90(0.44-1.82)	57	1.11(0.76-1.64)	33	0.78(0.49-1.24)	34	1.38(0.82-2.32)	55	0.90(0.62-1.30)	20	1.38(0.69-2.76)
	16	1.01(0.53-2.12)	57	1.24(0.84-1.82)	44	1.15(0.74-1.78)	24	1.10(0.63-1.94)	66	1.18(0.83-1.68)	13	1.07(0.50-2.30)
*p-trend*		*0.972*		*0.220*		*0.816*		*0.488*		*0.550*		*0.525*

Adjusted for age, level of education, smoking habits and alcohol consumption.

### Relative risk of colon versus rectal cancer

Separate analysis of risk of colon and rectal cancer demonstrated an overall increased risk of colon cancer for all anthropometric factors except for bodyfat percentage (Tables 6 and 7). Weight, hip, waist and BMI were significantly associated with an increased risk of rectal cancer. Among men, there was an increased risk of colon but not rectal cancer. In women, no association could be found between anthropometric factors and colon cancer risk, but a statistically significant association was seen between weight, bodyfat, hip, and waist and risk of rectal cancer (Tables 6 and 7).

**Table 6:** Anthropometric factors and relative risk of colon cancer in the full cohort, women and men

**Anthropometric factor (quartiles)**	**(n)**	**RR all n=337**	**(n)**	**RR men n=157**	**(n)**	**RR women n=180**
Height	76	1.00	32	1.00	37	1.00
	77	1.02(0.74-1.40)	37	1.27(0.79-2.04)	59	1.23(0.81-1.86)
	82	1.10(0.80-1.52)	40	1.07(0.67-1.72)	44	1.31(0.84-2.04)
	102	1.37(1.00-1.88)	48	1.52(0.96-2.42)	40	1.33(0.83-2.12)
*p-trend*		*0.041*		*0.137*		*0.221*
Weight	65	1.00	27	1.00	31	1.00
	72	1.11(0.79-1.56)	38	1.33(0.81-2.19)	43	1.18(0.74-1.88)
	91	1.23(0.89-1.70)	39	1.40(0.86-2.30)	51	1.59(1.01-2.00)
	109	1.51(1.10-2.07)	53	1.96(1.22-3.14)	55	1.39(0.89-2.17)
*p-trend*		*0.007*		*0.005*		*0.088*
Body fat percentage	72	1.00	24	1.00	30	1.00
	84	1.09(0.79-1.49)	34	1.10(0.65-1.86)	38	1.14(0.70-1.85)
	77	1.00(0.72-1.39)	44	1.05(0.64-1.73)	44	1.15(0.72-1.83)
	107	1.04(0.76-1.43)	54	1.64(1.01-2.67)	68	1.19(0.77-1.85)
*p-trend*		*0.880*		*0.039*		*0.470*
Hip	59	1.00	29	1.00	30	1.00
	66	0.92(0.65-1.31)	23	0.74(0.43-1.28)	39	0.89(0.55-1.43)
	99	1.31(0.94-1.82)	49	1.51(0.95-2.40)	54	1.15(0.73-1.82)
	113	1.23(0.89-1.70)	56	1.42(0.90-2.24)	68	1.00(0.63-1.57)
*p-trend*		*0.049*		*0.019*		*0.725*
Waist	60	1.00	27	1.00	31	1.00
	76	1.12(0.79-1.58)	22	0.72(0.41-1.27)	38	0.83(0.51-1.35)
	71	0.92(0.65-1.31)	51	1.57(0.98-2.50)	58	1.15(0.74-1.79)
	130	1.63(1.18-2.24)	57	1.84(1.16-2.92)	53	0.98(0.62-1.55)
*p-trend*		*0.003*		*<0.001*		*0.671*
Bmi	56	1.00	33	1.00	30	1.00
	65	1.13(0.79-1.63)	29	0.85(0.51-1.40)	40	1.13(0.70-1.82)
	106	1.39(1.00-1.93)	40	1.10(0.69-1.76)	57	1.52(0.97-2.37)
	110	1.61(1.16-2.24)	55	1.78(1.14-2.74)	53	1.33(084-2.10)
*p-trend*		*0.001*		*0.004*		*0.134*
Whr	73	1.00	32	1.00	48	1.00
	77	1.05(0.76-1.45)	24	1.06(0.63-1.81)	33	0.85(0.54-1.32)
	75	0.96(0.69-1.33)	52	1.84(1.18-2.87)	51	1.10(0.71-1.58)
	112	1.61(1.18-2.20)	49	2.01(1.28-3.16)	48	1.9(0.72-1.64)
*p-trend*		*0.005*		*<0.001*		*0.509*

Adjusted for age, level of education, smoking habits and alcohol consumption.

**Table 7:** Anthropometric factors and relative risk of rectal cancer in the full cohort, women and men

**Anthropometric factor (quartiles)**	**(n)**	**RR all n=213**	**(n)**	**RR men n=107**	**(n)**	**RR women n=106**
Height	46	1.00	26	1.00	19	1.00
	46	0.93(0.61-1.40)	27	1.13(0.66-1.93)	37	1.36(0.78-2.37)
	61	1.22(0.83-1.81)	28	0.93(0.55-1.60)	27	1.31(0.72-2.37)
	61	1.16(0.78-1.74)	26	1.03(0.59-1.80)	23	1.18(0.63-2.21)
*p-trend*		*0.273*		*0.902*		*0.713*
Weight	37	1.00	24	1.00	12	1.00
	52	1.45(0.95-2.21)	26	1.04(0.60-1.82)	33	2.36(1.22-4.58)
	54	1.27(0.83-1.94)	26	1.06(0.60-1.85)	27	2.34(1.18-4.62)
	71	1.68(1.12-2.53)	31	1.26(0.73-2.16)	34	2.46(1.27-4.77)
*p-trend*		*0.029*		*0.411*		*0.022*
Body fatper centage	51	1.00	15	1.00	15	1.00
	55	1.03(0.70-1.50)	19	0.98(0.50-1.94)	18	1.26(0.63-2.51)
	38	0.73(0.48-1.12)	44	1.64(0.91-2.95)	33	2.05(1.10-3.80)
	70	1.05(0.72-1.53)	28	1.31(0.70-2.47)	40	1.83(1.00-3.37)
*p-trend*		*0.956*		*0.173*		*0.026*
Hip	33	1.00	17	1.00	13	1.00
	43	1.13(0.72-1.78)	26	1.48(0.80-2.73)	26	1.52(0.78-2.97)
	61	1.59(103-2.44)	31	1.71(0.94-3.10)	24	1.45(0.74-2.88)
	77	1.69(1.12-2.57)	33	1.52(0.84-2.76)	43	2.30(1.22-4.34)
*p-trend*		*0.003*		*0.180*		*0.009*
Waist	31	1.00	23	1.00	10	1.00
	53	1.50(0.96-2.34)	18	0.70(0.38-1.30)	29	2.24(1.09-4.60)
	48	1.21(0.76-1.91)	32	1.14(0.67-1.96)	34	2.38(1.17-4.83)
	82	1.96(1.27-3.00)	34	1.26(0.74-2.169	33	2.43(1.19-4.98)
*p-trend*		*0.005*		*0.160*		*0.032*
Bmi	39	1.00	30	1.00	15	1.00
	50	1.31(0.86-1.99)	18	0.57(0.32-1.03)	35	2.22(1.21-4.08)
	51	0.99(0.65-1.51)	24	0.72(0.42-1.23)	22	1.34(0.69-2.61)
	74	1.62(1.09-2.42)	35	1.16(0.70-1.91)	34	2.17(1.17-4.04)
*p-trend*		*0.044*		*0.407*		*0.093*
Whr	44	1.00	33	1.00	29	1.00
	43	0.96(0.63-1.47)	21	0.86(0.50-1.49)	28	1.36(0.79-2.34)
	64	1.31(0.89-1.94)	24	0.77(0.45-1.31)	25	1.02(0.58-1.79)
	62	1.32(0.88-1.99)	29	1.04(0.62-1.72)	29	1.35(0.79-2.34)
*p-trend*		*0.082*		*0.952*		*0.475*

Adjusted for age, level of education, smoking habits and alcohol consumption.

Among women, all anthropometric factors except height and WHR were significantly associated with risk of a more advanced T-stage in rectal but not colon cancer, while there were no associations with risk of more advanced N-stage, neither in colon nor rectal cancer (data not shown). In addition, there was a significant association between height and M1 disease in colon but not rectal cancer in women (data not shown). In men, there were no associations between anthropometric factors and risk of more advanced T-stage in rectal cancer, but in colon cancer, only height was associated with a statistically significant increased risk of T-stage 3 & 4 disease (data not shown). Moreover, among men, there was a statistically significant increased risk of developing N1-2 tumours related to height, weight, waist and hip in colon and to hip, BMI and WHR in rectal cancer, and a statistically significant association between BMI and M1 disease in colon but not rectum (data not shown).

## Discussion

The results from this large prospective cohort study indicate that the association between obesity and risk of CRC varies by gender, cancer site and tumour aggressiveness. All anthropometric factors except bodyfat percentage were significantly associated with an increased risk of CRC in general, which corresponds well to previous findings [3,4,6,12,13,15,18,19,32]. When studying men and women separately, we found an increased risk of CRC for all anthropometric variables, except for height, in men, which is also consistent with previous results [4-6,12,15,16]. However, the finding of a stronger association of obesity and risk of being diagnosed with clinically more advanced CRC in men has, to our knowledge, not been demonstrated before. We also found an overall increased risk of CRC with obesity defined as weight, BMI and WHR in women, which has only been shown in a few previous studies [4,10,15,18,20].

To our knowledge, only two previous studies [13,18] have examined the associations between anthropometric factors and risk of CRC according to tumour aggressiveness, as reflected in TNM-classification of the disease. These two studies examined the association between anthropometry and risk of colon cancer according to late (Stage III and IV) vs early (Stage I and II) stage colon cancer in men and women, respectively, and neither found any statistically significant associations.

We have thus been able to demonstrate an increased risk of more advanced tumours with increased anthropometric measures, particularly in men, among whom several anthropometric variables were significantly associated with risk of a more advanced T-stage, N2 disease, and both M0 and M1-tumours. In women, several anthropometric variables were associated with an increased risk of more advanced T-stage, but not node-positive disease. Furthermore, increased anthropometric measures were only associated with non-metastatic (M0) disease in women. Taken together, these findings suggest that overweight persons have an increased risk of developing more advanced colorectal cancer forms, but that this risk may differ according to gender.

Moreover, our findings provide further evidence of gender-related differences in tumour susceptibility of cancer in the colon and rectum, respectively, as we found a positive association between obesity and risk of developing colon cancer in men and rectal cancer in women. This has to our knowledge not been shown previously, since only a few studies have examined risk of colon and rectal cancer separately. However, two previous studies [4,14] have shown a positive relationship between BMI, overweight and rectal cancer risk in men, which is in contrast to our findings. Moreover, subgroup analysis related to risk of more advanced disease in colon and rectal cancer, respectively, also revealed a more pronounced association between anthropometric factors and risk of advanced disease in men.

Body weight or BMI have been the most commonly used anthropometric measurements to examine the associations of obesity and colorectal cancer risk in previous studies, the majority of which have shown a positive relationship between BMI and risk of CRC in men, but week or no associations in women [3-16]. However, these measurements may not be ideal because of the changes in physiologic functions that to a certain extent depend on regional adipose tissue distribution [33]. Some prospective studies have examined the association of body fat distribution, reflected as waist- and hip circumference, and colorectal cancer risk [6,8,12,13,18-22], but available epidemiologic evidence suggests that abdominal obesity (high waist circumference and waist-hip-ratio) may be more predictive of colorectal cancer risk than overall obesity [6,8,12,13,18]. Increased bodyweight has been suggested to be more closely related to abdominal obesity in men, and to gluteofemoral obesity in women [17].

The exact biologic mechanisms underlying the association between obesity and increased risk of CRC are not fully understood. Some authors have suggested components of the metabolic syndrome, in particular insulin-resistance and subsequent hyperinsulinemia, to be the underlying link, which may reflect the growth-promoting effects of insulin [32,34]. These speculations are also supported by studies that found an increased risk of CRC in patients with Type 2 diabetes [35-37], as hyperinsulinemia is also related to increased levels of insulin-like growth factor 1, which is known to have cancer promoting effects [38-40]. Further potential mediators include leptin, elevated serum levels of which have been demonstrated to stimulate growth of colonic epithelial cells [41-43], and adinopectin, that is secreted from visceral adipose tissue, correlates inversely to BMI, and has been demonstrated to have antiangionenic and antitumour activities [44,45]. Obesity, in particular abdominal obesity, is linked to insulin-resistance, hyperinsulinemia and Type 2 diabetes [46].

Several epidemiological studies have shown that elevated levels of estrogen and progesterone is associated with lower risk of developing CRC [47-49]. Recent studies found no reduced risk of CRC in women with higher levels of estradiol and estrone, suggesting that progesterone is the key factor for reduction of CRC risk in women [50-52]. Use of hormone replacement therapy (HRT) in post-menopausal women could in part explain sex-related differences in the association between adiposity and CRC risk, as BMI is positively associated with circulating levels of estradiol in postmenopausal women and HRT has been associated with a reduced risk of CRC in observational and interventional studies [53-56]. The associations between sex hormones and risk of CRC in men is poorly understood, but two studies have supported the hypothesis that lower androgenecity may increase men’s risk of developing CRC [57,58]. Lower androgen levels seem to be more frequent in obese men, and treatment with testosterone reduces insulin resistance, suggesting a role of androgens in promoting insuline sensitivity and hereby one possible mechanism in the development of CRC.

In agreement with most previous reports, we found a stronger association between body size and risk of colon compared to rectal cancer in general, in particular in men [4]. In line with these findings, a meta-analysis showed that physical activity, which is related to improved insulin sensitivity [59,60], was associated with a reduced risk of colon cancer, but not of rectal cancer [61]. This may suggest that insulin resistance, hyperinsulinemia and other factors related to obesity are stronger risk factors for colon than rectal cancer. Several prospective studies reported that circulating C-peptide [39,42,62,63] and leptin [42,43] concentrations were more strongly associated with risk of colon cancer than overall colorectal cancer or rectal cancer. Our findings of a significant association between high weight, bodyfat percentage, hip and waist, and an increased risk of rectal cancer in women but not men are in contrast to most previous studies, including a large meta-analysis demonstrating a statistically significant association between obesity and an increased risk of rectal cancer in men [4]. While these findings indeed suggest differences in tumour susceptibility related to location, the potential mechanisms accounting for these differences need further investigation.

A limitation to the present study is the relatively small number of cases in some subgroups, i.e. it is possible that some of the non-significant findings in relation to gender or tumour stages may have been caused by a potential type II error. Therefore, these data need to be validated in larger patient cohorts. It should also be pointed out that since mean age at diagnosis was >70 years for women [27], interpretations relying on sex as evidence for influence of sex hormones must be made with caution until further knowledge has been gained. To this end, future studies are warranted to explore the effects of e.g. hormone replacement therapy on clinicopathological factors, molecular correlates and survival from CRC.

Certain methodological aspects need further attention. The validity of the anthropometric measurements is one aspect as there may be a potential inter-observer variation. Recommendations for the nurses performing baseline examinations described how participants should be dressed, in which position the participants should be examined, and location for the estimation of waist- and hip measurements. We therefore consider the risk of misclassification of anthropometric measurements to be low. In contrast, most previous studies have used self-reported anthropometric measures.

It is also possible that participation in the MDCS was associated with body constitution, which may have lead to potential selection bias. In a previous paper, Manjer et al [25] compared BMI in the MDCS in relation to the background population, and found an equal distribution of overweight/obesity.

Another aspect is the validity of collected data. As anthropometric data was assessed only at baseline, it is possible that some individuals have gained and some have lost weight. Such a misclassification is likely to lead to an attenuation of risks and, if anything, observed risks may be underestimated.

In addition, it might be difficult to apply incidence rates from the MDCS to the background population as participants may to some degree have been selected in terms of socio-economic factors and risk of CRC. Nevertheless, we consider it possible to make internal comparisons comparing subjects with high versus low levels of the study measurements in order to obtain relative risks.

## Conclusion

This study is the first to show a relationship between obesity, measured as several different anthropometric factors, and an increased risk of colorectal cancer of more advanced clinical stage, in particular in men. These findings need to be confirmed in future studies, as they may be of importance in the development of novel strategies for prevention of the disease, such as tailored screening programmes taking sex and anthropometric factors into account.

## Abbreviations

CRC: Colorectal cancer; MDCS: Malmö diet and cancer study; BMI: Body mass index; WHR: Waist-hip ratio; T-stage: Tumour stage; N-stage: Nodal stage; M-stage: Metastatic stage; HRT: Hormone replacement therapy.

## Competing interests

The authors declare that no competing interests exist.

## Authors’ contributions

JB performed the statistical analyses and drafted the manuscript. SW, BN and AG collected clinical data. JM assisted with the statistical analyses and helped draft the manuscript KJ conceived of the study, carried out the histopathological re-evaluation, and helped draft the manuscript. All authors read and approved the final manuscript.

## Supplementary Material

Additional file 1 Distribution of quartiles of anthropometric measurements. Click here for file

## References

[B1] Parkin DM Bray F Ferlay J Pisani P Global cancer statistics, 2002 CA Cancer J Clin 2005 55 2 74 108 10.3322/canjclin.55.2.74 15761078

[B2] Eheman C Henley SJ Ballard-Barbash R Jacobs EJ Schymura MJ Noone AM Pan L Anderson RN Fulton JE Kohler BA Annual Report to the Nation on the status of cancer, 1975-2008, featuring cancers associated with excess weight and lack of sufficient physical activity Cancer 2012 118 9 2338 2366 10.1002/cncr.27514 22460733 PMC4586174

[B3] Dai Z Xu YC Niu L Obesity and colorectal cancer risk: a meta-analysis of cohort studies World J Gastroenterol 2007 13 31 4199 4206 17696248 10.3748/wjg.v13.i31.4199 PMC4250618

[B4] Larsson SC Wolk A Obesity and colon and rectal cancer risk: a meta-analysis of prospective studies Am J Clin Nutr 2007 86 3 556 565 17823417 10.1093/ajcn/86.3.556

[B5] Renehan AG Tyson M Egger M Heller RF Zwahlen M Body-mass index and incidence of cancer: a systematic review and meta-analysis of prospective observational studies Lancet 2008 371 9612 569 578 10.1016/S0140-6736(08)60269-X 18280327

[B6] Pischon T Lahmann PH Boeing H Friedenreich C Norat T Tjonneland A Halkjaer J Overvad K Clavel-Chapelon F Boutron-Ruault MC Body size and risk of colon and rectal cancer in the European Prospective Investigation Into Cancer and Nutrition (EPIC) J Natl Cancer Inst 2006 98 13 920 931 10.1093/jnci/djj246 16818856

[B7] Terry PD Miller AB Rohan TE Obesity and colorectal cancer risk in women Gut 2002 51 2 191 194 10.1136/gut.51.2.191 12117878 PMC1773328

[B8] Giovannucci E Ascherio A Rimm EB Colditz GA Stampfer MJ Willett WC Physical activity, obesity, and risk for colon cancer and adenoma in men Ann Intern Med 1995 122 5 327 334 7847643 10.7326/0003-4819-122-5-199503010-00002

[B9] Murphy TK Calle EE Rodriguez C Kahn HS Thun MJ Body mass index and colon cancer mortality in a large prospective study Am J Epidemiol 2000 152 9 847 854 10.1093/aje/152.9.847 11085396

[B10] Lin J Zhang SM Cook NR Rexrode KM Lee IM Buring JE Body mass index and risk of colorectal cancer in women (United States) Cancer Causes Control 2004 15 6 581 589 15280637 10.1023/B:CACO.0000036168.23351.f1

[B11] Terry P Giovannucci E Bergkvist L Holmberg L Wolk A Body weight and colorectal cancer risk in a cohort of Swedish women: relation varies by age and cancer site Br J Cancer 2001 85 3 346 349 11487263 10.1054/bjoc.2001.1894 PMC2364077

[B12] Moore LL Bradlee ML Singer MR Splansky GL Proctor MH Ellison RC Kreger BE BMI and waist circumference as predictors of lifetime colon cancer risk in Framingham Study adults Int J Obes Relat Metab Disord 2004 28 4 559 567 10.1038/sj.ijo.0802606 14770200

[B13] MacInnis RJ English DR Hopper JL Haydon AM Gertig DM Giles GG Body size and composition and colon cancer risk in men Cancer Epidemiol Biomarkers Prev 2004 13 4 553 559 15066919

[B14] Kune GA Kune S Watson LF Body weight and physical activity as predictors of colorectal cancer risk Nutr Cancer 1990 13 1–2 9 17 2300499 10.1080/01635589009514041

[B15] Caan BJ Coates AO Slattery ML Potter JD Quesenberry SM Jr Edwards CP Body size and the risk of colon cancer in a large case-control study Int J Obes Relat Metab Disord 1998 22 2 178 184 10.1038/sj.ijo.0800561 9504326

[B16] Russo A Franceschi S La Vecchia C Dal Maso L Montella M Conti E Giacosa A Falcini F Negri E Body size and colorectal-cancer risk Int J Cancer 1998 78 2 161 165 10.1002/(SICI)1097-0215(19981005)78:2<161::AID-IJC7>3.0.CO;2-X 9754646

[B17] Krotkiewski M Bjorntorp P Sjostrom L Smith U Impact of obesity on metabolism in men and women. Importance of regional adipose tissue distribution J Clin Invest 1983 72 3 1150 1162 10.1172/JCI111040 6350364 PMC1129283

[B18] MacInnis RJ English DR Hopper JL Gertig DM Haydon AM Giles GG Body size and composition and colon cancer risk in women Int J Cancer 2006 118 6 1496 1500 10.1002/ijc.21508 16187280

[B19] Schoen RE Tangen CM Kuller LH Burke GL Cushman M Tracy RP Dobs A Savage PJ Increased blood glucose and insulin, body size, and incident colorectal cancer J Natl Cancer Inst 1999 91 13 1147 1154 10.1093/jnci/91.13.1147 10393723

[B20] Martinez ME Giovannucci E Spiegelman D Hunter DJ Willett WC Colditz GA Leisure-time physical activity, body size, and colon cancer in women Nurses’ Health Study Research Group. J Natl Cancer Inst 1997 89 13 948 955 10.1093/jnci/89.13.948 9214674

[B21] Folsom AR Kushi LH Anderson KE Mink PJ Olson JE Hong CP Sellers TA Lazovich D Prineas RJ Associations of general and abdominal obesity with multiple health outcomes in older women: the Iowa Women’s Health Study Arch Intern Med 2000 160 14 2117 2128 10.1001/archinte.160.14.2117 10904454

[B22] Larsson SC Rutegard J Bergkvist L Wolk A Physical activity, obesity, and risk of colon and rectal cancer in a cohort of Swedish men Eur J Cancer 2006 42 15 2590 2597 10.1016/j.ejca.2006.04.015 16914307

[B23] Bostick RM Potter JD Kushi LH Sellers TA Steinmetz KA McKenzie DR Gapstur SM Folsom AR Sugar, meat, and fat intake, and non-dietary risk factors for colon cancer incidence in Iowa women (United States) Cancer Causes Control 1994 5 1 38 52 10.1007/BF01830725 8123778

[B24] Riboli E Kaaks R The EPIC Project: rationale and study design. European Prospective Investigation into Cancer and Nutrition Int J Epidemiol 1997 26 Suppl 1 S6 S14 9126529 10.1093/ije/26.suppl_1.s6

[B25] Manjer J Carlsson S Elmstahl S Gullberg B Janzon L Lindstrom M Mattisson I Berglund G The Malmo Diet and Cancer Study: representativity, cancer incidence and mortality in participants and non-participants Eur J Cancer Prev 2001 10 6 489 499 10.1097/00008469-200112000-00003 11916347

[B26] Steen B Bosaeus I Elmstahl S Galvard H Isaksson B Robertsson E Body composition in the elderly estimated with an electrical impedance method Compr Gerontol A 1987 1 3 102 105 3453289

[B27] Wangefjord S Manjer J Gaber A Nodin B Eberhard J Jirstrom K Cyclin D1 expression in colorectal cancer is a favorable prognostic factor in men but not in women in a prospective, population-based cohort study Biol Sex Differ 2011 2 10 10.1186/2042-6410-2-10 21888663 PMC3179695

[B28] Larsson A Johansson ME Wangefjord S Gaber A Nodin B Kucharzewska P Welinder C Belting M Eberhard J Johnsson A Overexpression of podocalyxin-like protein is an independent factor of poor prognosis in colorectal cancer Br J Cancer 2011 105 5 666 672 10.1038/bjc.2011.295 21829192 PMC3188928

[B29] Eberhard J Gaber A Wangefjord S Nodin B Uhlén M Ericson Lindquist K Jirström K A cohort study of the prognostic and treatment predictive value of SATB2 expression in colorectal cancer Br J Cancer 2012 106 5 931 938 10.1038/bjc.2012.34 22333599 PMC3305956

[B30] Nodin B Johannesson H Wangefjord S DP OC Ericson-Lindquist K Uhlen M Jirstrom K Eberhard J Molecular correlates and prognostic significance of SATB1 expression in colorectal cancer Diagn Pathol 2012 7 1 115 10.1186/1746-1596-7-115 22935204 PMC3523011

[B31] Katz MH Hauck WW Proportional hazards (Cox) regression J Gen Intern Med 1993 8 12 702 711 10.1007/BF02598295 8120690

[B32] Giovannucci E Diet, body weight, and colorectal cancer: a summary of the epidemiologic evidence J Womens Health (Larchmt) 2003 12 2 173 182 10.1089/154099903321576574 12737716

[B33] Karastergiou K Fried SK Sex differences in human adipose tissues - the biology of pear shape Biol Sex Differ 2012 3 1 13 10.1186/2042-6410-3-13 22651247 PMC3411490

[B34] Giovannucci E Insulin, insulin-like growth factors and colon cancer: a review of the evidence J Nutr 2001 131 11 Suppl 3109S 3120S 11694656 10.1093/jn/131.11.3109S

[B35] Hu FB Manson JE Liu S Hunter D Colditz GA Michels KB Speizer FE Giovannucci E Prospective study of adult onset diabetes mellitus (type 2) and risk of colorectal cancer in women J Natl Cancer Inst 1999 91 6 542 547 10.1093/jnci/91.6.542 10088625

[B36] McKeown-Eyssen G Epidemiology of colorectal cancer revisited: are serum triglycerides and/or plasma glucose associated with risk? Cancer Epidemiol Biomarkers Prev 1994 3 8 687 695 7881343

[B37] Seow A Yuan JM Koh WP Lee HP Yu MC Diabetes mellitus and risk of colorectal cancer in the Singapore Chinese Health Study J Natl Cancer Inst 2006 98 2 135 138 10.1093/jnci/djj015 16418516

[B38] Aaronson SA Growth factors and cancer Science 1991 254 5035 1146 1153 10.1126/science.1659742 1659742

[B39] Kaaks R Toniolo P Akhmedkhanov A Lukanova A Biessy C Dechaud H Rinaldi S Zeleniuch-Jacquotte A Shore RE Riboli E Serum C-peptide, insulin-like growth factor (IGF)-I, IGF-binding proteins, and colorectal cancer risk in women J Natl Cancer Inst 2000 92 19 1592 1600 10.1093/jnci/92.19.1592 11018095

[B40] Sandhu MS Dunger DB Giovannucci EL Insulin, insulin-like growth factor-I (IGF-I), IGF binding proteins, their biologic interactions, and colorectal cancer J Natl Cancer Inst 2002 94 13 972 980 10.1093/jnci/94.13.972 12096082

[B41] Hardwick JC Van Den Brink GR Offerhaus GJ Van Deventer SJ Peppelenbosch MP Leptin is a growth factor for colonic epithelial cells Gastroenterology 2001 121 1 79 90 10.1053/gast.2001.25490 11438496

[B42] Stattin P Lukanova A Biessy C Soderberg S Palmqvist R Kaaks R Olsson T Jellum E Obesity and colon cancer: does leptin provide a link? Int J Cancer 2004 109 1 149 152 10.1002/ijc.11668 14735482

[B43] Stattin P Palmqvist R Soderberg S Biessy C Ardnor B Hallmans G Kaaks R Olsson T Plasma leptin and colorectal cancer risk: a prospective study in Northern Sweden Oncol Rep 2003 10 6 2015 2021 14534736

[B44] Brakenhielm E Veitonmaki N Cao R Kihara S Matsuzawa Y Zhivotovsky B Funahashi T Cao Y Adiponectin-induced antiangiogenesis and antitumor activity involve caspase-mediated endothelial cell apoptosis Proc Natl Acad Sci USA 2004 101 8 2476 2481 10.1073/pnas.0308671100 14983034 PMC356975

[B45] Wei EK Giovannucci E Fuchs CS Willett WC Mantzoros CS Low plasma adiponectin levels and risk of colorectal cancer in men: a prospective study J Natl Cancer Inst 2005 97 22 1688 1694 10.1093/jnci/dji376 16288122

[B46] Kahn BB Flier JS Obesity and insulin resistance J Clin Invest 2000 106 4 473 481 10.1172/JCI10842 10953022 PMC380258

[B47] La Vecchia C Franceschi S Reproductive factors and colorectal cancer Cancer Causes Control 1991 2 3 193 200 10.1007/BF00056213 1873449

[B48] Fernandez E La Vecchia C Balducci A Chatenoud L Franceschi S Negri E Oral contraceptives and colorectal cancer risk: a meta-analysis Br J Cancer 2001 84 5 722 727 10.1054/bjoc.2000.1622 11237397 PMC2363788

[B49] Grodstein F Newcomb PA Stampfer MJ Postmenopausal hormone therapy and the risk of colorectal cancer: a review and meta-analysis Am J Med 1999 106 5 574 582 10.1016/S0002-9343(99)00063-7 10335731

[B50] Gunter MJ Hoover DR Yu H Wassertheil-Smoller S Rohan TE Manson JE Howard BV Wylie-Rosett J Anderson GL Ho GY Insulin, insulin-like growth factor-I, endogenous estradiol, and risk of colorectal cancer in postmenopausal women Cancer Res 2008 68 1 329 337 10.1158/0008-5472.CAN-07-2946 18172327 PMC4225702

[B51] Clendenen TV Koenig KL Shore RE Levitz M Arslan AA Zeleniuch-Jacquotte A Postmenopausal levels of endogenous sex hormones and risk of colorectal cancer Cancer Epidemiol Biomarkers Prev 2009 18 1 275 281 10.1158/1055-9965.EPI-08-0777 19124509 PMC2682428

[B52] Ritenbaugh C Stanford JL Wu L Shikany JM Schoen RE Stefanick ML Taylor V Garland C Frank G Lane D Conjugated equine estrogens and colorectal cancer incidence and survival: the Women’s Health Initiative randomized clinical trial Cancer Epidemiol Biomarkers Prev 2008 17 10 2609 2618 10.1158/1055-9965.EPI-08-0385 18829444 PMC2937217

[B53] Nelson HD Humphrey LL Nygren P Teutsch SM Allan JD Postmenopausal hormone replacement therapy: scientific review JAMA 2002 288 7 872 881 10.1001/jama.288.7.872 12186605

[B54] Rossouw JE Anderson GL Prentice RL LaCroix AZ Kooperberg C Stefanick ML Jackson RD Beresford SA Howard BV Johnson KC Risks and benefits of estrogen plus progestin in healthy postmenopausal women: principal results From the Women’s Health Initiative randomized controlled trial JAMA 2002 288 3 321 333 10.1001/jama.288.3.321 12117397

[B55] Anderson GL Limacher M Assaf AR Bassford T Beresford SA Black H Bonds D Brunner R Brzyski R Caan B Effects of conjugated equine estrogen in postmenopausal women with hysterectomy: the Women’s Health Initiative randomized controlled trial JAMA 2004 291 14 1701 1712 15082697 10.1001/jama.291.14.1701

[B56] Chlebowski RT Wactawski-Wende J Ritenbaugh C Hubbell FA Ascensao J Rodabough RJ Rosenberg CA Taylor VM Harris R Chen C Estrogen plus progestin and colorectal cancer in postmenopausal women N Engl J Med 2004 350 10 991 1004 10.1056/NEJMoa032071 14999111

[B57] Slattery ML Sweeney C Murtaugh M Ma KN Wolff RK Potter JD Caan BJ Samowitz W Associations between ERalpha, ERbeta, and AR genotypes and colon and rectal cancer Cancer Epidemiol Biomarkers Prev 2005 14 12 2936 2942 10.1158/1055-9965.EPI-05-0514 16365013

[B58] Alberg AJ Gordon GB Hoffman SC Comstock GW Helzlsouer KJ Serum dehydroepiandrosterone and dehydroepiandrosterone sulfate and the subsequent risk of developing colon cancer Cancer Epidemiol Biomarkers Prev 2000 9 5 517 521 10815698

[B59] Houmard JA Tanner CJ Slentz CA Duscha BD McCartney JS Kraus WE Effect of the volume and intensity of exercise training on insulin sensitivity J Appl Physiol 2004 96 1 101 106 12972442 10.1152/japplphysiol.00707.2003

[B60] Ivy JL Role of exercise training in the prevention and treatment of insulin resistance and non-insulin-dependent diabetes mellitus Sports Med 1997 24 5 321 336 10.2165/00007256-199724050-00004 9368278

[B61] Samad AK Taylor RS Marshall T Chapman MA A meta-analysis of the association of physical activity with reduced risk of colorectal cancer Colorectal Dis 2005 7 3 204 213 10.1111/j.1463-1318.2005.00747.x 15859955

[B62] Shimizu N Nagata C Shimizu H Kametani M Takeyama N Ohnuma T Matsushita S Height, weight, and alcohol consumption in relation to the risk of colorectal cancer in Japan: a prospective study Br J Cancer 2003 88 7 1038 1043 10.1038/sj.bjc.6600845 12671701 PMC2376385

[B63] Jenab M Riboli E Cleveland RJ Norat T Rinaldi S Nieters A Biessy C Tjonneland A Olsen A Overvad K Serum C-peptide, IGFBP-1 and IGFBP-2 and risk of colon and rectal cancers in the European Prospective Investigation into Cancer and Nutrition Int J Cancer 2007 121 2 368 376 10.1002/ijc.22697 17372899

