# Supplementary material for: Gender, anthropometric factors and risk of colorectal cancer with particular reference to tumour location and TNM stage: a cohort study
Source: Biol Sex Differ. 2012 Oct 16;3:23. doi: 10.1186/2042-6410-3-23 (PMC3504577; doi:10.1186/2042-6410-3-23)
Supplement: Additional file 1 — Distribution of quartiles of anthropometric measurements. [file 2042-6410-3-23-S1.doc]

**Additional file 1. Distribution of quartiles of anthropometric measurements**

| Quartiles | All | Women | Men |
| --- | --- | --- | --- |
| Height (cm) | <162  ≥162-<168  ≥168-<175  ≥175 | <159  ≥159-<164  ≥164-<168  ≥168 | <172  ≥172-<176  ≥176-<181  ≥181 |
| Weight (kg) | <64  ≥64-<72  ≥72-<82  ≥82 | <60  ≥60-<67  ≥67-<74  ≥74 | <74  ≥74-<81  ≥81-<89  ≥89 |
| Bodyfatpercentage(%) | <21  ≥21-<27  ≥27-<32  ≥32 | <28  ≥28-<31  ≥31-<34  ≥34 | <17  ≥17-<20  ≥20-<24  ≥24 |
| Hip (cm) | <74  ≥74-<83  ≥83-<93  ≥93 | <91  ≥91-<97  ≥97-<103  ≥103 | <95  ≥95-<99  ≥99-<103  ≥103 |
| Waist (cm) | <93  ≥93-<98  ≥98-<103  ≥103 | <70  ≥70-<76  ≥76-<84  ≥84 | <87  ≥87-<93  ≥93<100  ≥100 |
| BMI (kg/m²) | <23  ≥23-<25  ≥25-<28  ≥28 | <22  ≥22-<25  ≥25-<28  ≥28 | <24  ≥24-<26  ≥26-<28  ≥28 |
| WHR (cm/cm) | <0.78  ≥0.78-<0.84  ≥0.84-<0.93  ≥0.93 | <0.76  ≥0.76-<0.79  ≥0.79-<0.83  ≥0.83 | <0.91  ≥0.91-<0.94  ≥0.94-<0.98  ≥0.98 |
